# Editorial: Immunocellular mechanisms and endocrine organs

**DOI:** 10.3389/fendo.2023.1214230

**Published:** 2023-05-16

**Authors:** Daniela Gallo, Yusuf Ali

**Affiliations:** ^1^Endocrine Unit, Azienda Socio Sanitaria dei Sette Laghi (ASST), Varese, Italy; ^2^Lee Kong Chian School of Medicine, Nanyang Technological University, Singapore, Singapore

**Keywords:** niche innate immune cells, cytokines in endocrine disorders, drivers of endocrine tissue remodelling, thyroid, aging, macrophages, natural killer cells, endometriosis

Immune cells play multiple roles that go beyond mere pathogen defense, and this is certainly true for cells of the innate immune system. Innate immune cells circulate in the bloodstream and are present in various tissue niches acting as surveillance sentinels and first responders against foreign substances and insults such as tissue damage (1). Arguably, innate immune cells can mediate tissue crosstalk and contribute to endocrine feedback loops to maintain tissue as well as whole body homeostasis, and vice versa their activity can be regulated by hormones too, putting them squarely as endocrine mediators. Certain conditions escalate innate cell response leading to acquired immune reactions directed against self-antigens in endocrine organs. This Research Topic delves into the role of innate immunity in the pathogenesis and treatment of endocrine-related disorders and the crosstalk between innate immunity, hormone secretion and metabolic homeostasis. We collated a series of interesting original research and review articles that together represent a vignette of innate immune cells’ contribution to chronic endocrine diseases such as endometriosis type-2 diabetes, post-traumatic inflammation (bone fracture), autoimmune thyroid disorders (AITD) and thyroid carcinomas.

Endometriosis (EMS) is a chronic disease, which impairs patients’ quality of life through dysmenorrhea, chronic pelvic pain, and infertility (2, 3). Accumulating evidence supports the hypothesis that EMS is the result of a local aseptic inflammation, sustained by various immune cells including activated macrophages. The diagnosis is often delayed and needs the application of invasive techniques; thus, the identification of reliable peripheral biomarkers is crucial (3). By combining data from EMS-related human genomic databases, Yang and coworkers identified 332 differentially expressed genes (DEGs) associated with endometriosis, most of which are involved in the regulation of immune homeostasis and cell proliferation and migration (integrins, adipokines, complement activation, signalling pathways participating in cell cycle). Bioinformatics analysis (LASSO machine-learning algorithm) identified five genes with diagnostic value (Receiver Operating Curve, ROC analysis) that were upregulated in ectopic endometrial tissue and were related to macrophage activation and a myriad of other immune cell functions.

HBCB-1 (High mobility group box 1) is a non-histone DNA binding protein secreted by several types of innate immune cells including dendritic cells, monocytes, macrophages and neutrophils which acts *via* Toll-like Receptor (TLR) 2 and TLR4 to mediate inflammation (4). Huang et al. observed that HMCG-1 expression positively correlates with inflammatory cytokines (interleukin-6 IL-6, IL-1β and Tumor Necrosis Factor-alpha TNFα) in endometriosis and that markers of autophagy including beclin-1 were increased in ectopic endometrium. HMGB-1 knockdown suppressed both inflammatory cytokines and beclin-1, suggesting a pathogenetic role of HMGB-1 in EMS.

In the study by Tylicka et al., HMGB-1 along with HSP-70, IL-6, C Reactive Protein (CRP) was used as precocious marker of local inflammatory damage after forearm distal fracture. Levels of HMGB-1 were subsequently used as a proxy for site inflammation monitoring following Kirschner wire fixation, which showed no inflammatory flare-up post-procedure.

With the aim of exploring biomarkers and novel diagnostic tools for human disorders, Chiari et al. provided an overview of the potential implications of long pentraxin 3 (PTX3) in benign (thyroid nodules, Graves’ disease) and malignant thyroid diseases. PTX3 is produced by neutrophils during their maturation in the bone marrow and acts as a soluble pattern recognition molecule, regulating complement cascade, tumor microenvironment, and tissue remodelling. In the thyroid gland follicular cells, resident immune cells, and extracellular matrix express PTX3, whose levels are increased in thyroid benign nodules and anaplastic cancer, along with fibroblasts of patients with Graves’ orbitopathy.

The available research on the pathogenesis of autoimmune disorders (AITD) is mostly devoted to adaptive immunity, but growing evidence suggests that innate immune cells also contribute to the initiation and perpetuation of inflammation. Additionally, treatments targeting the underlying immune response in AITD are lacking. Gallo et al. observed that in newly diagnosed Graves’ disease patients, the frequency of natural killer (NK) cells, especially of CD56^bright^ NK cell subset, was higher than in healthy controls, with a significantly higher proportion of NK cells expressing the activating receptor CD69. Because NK cells have immune regulatory functions but are impaired in hyperthyroidism (5, 6), these findings may reflect an unsuccessful attempt by NK cells to constrain inflammation at the very beginning, as supported by the lower degranulation ability. Randomizing 42 patients to moderate doses of selenium and cholecalciferol supplementation combined with standard anti-thyroid drugs treatment or anti-thyroid drugs alone, Gallo et al. observed that in the supplemented group total NK and NK cell subsets had a greater decrease during treatment compared to standard monotherapy, while the frequency of circulating T regulatory cells significantly increased. The combined treatment determined a faster and greater control of hyperthyroidism (7).

Pan et al. describe the effect of the Chinese drug JiaYanKangTai in a mouse model of chronic thyroiditis. The Authors demonstrated that JiaYanKangTai drug was able to decrease circulating levels of anti-thyroglobulin and anti-peroxidase antibodies and thyroid infiltration, by downregulating the expression of IL-17A, TNF receptor-associated factor (TRAF) 6, p-ERK1/2 and tumour necrosis factor-alpha.

Ma and Ruedl studied the kinetics of pancreatic resident macrophages in obesity and type-2 diabetes using an adult myeloid cell fate-mapping mouse model. Resident macrophages physiologically occupy tissue niches differently and their turnover, relative cell numbers and activation states are further altered in various pathologies (8, 9). The Authors observed that with increasing age intra-acinar embryonic macrophages (F4/80^hi^Tim-4^+^-MHCII^+^ macrophages) were replaced by bone marrow (BM)-derived monocytes, whereas the exocrine F4/80^hi^Tim-4^+^ subset was persistent and independent from BM-derived monocytes. However, turnover kinetics in the exocrine compartment of the pancreas was not influenced by obesity and type 2 diabetes.

To summarize, there is a complex intertwined relationship between the innate immune system and the endocrine system, which together function to maintain tissue and whole-body homeostasis. Further investigations that compare disease and healthy tissue states start to piece together this intricate crosstalk puzzle and we certainly hope that the Reader will find this Research Topic a useful resource in the field of immune and endocrine systems interaction.

## Author contributions

YA conceived the Research Topic. DG and YA served as Guest Editors of the Research Topic, wrote the manuscript and approved its final version.
